# Rethinking Healthspan: Beyond the Absence of Disease

**DOI:** 10.1093/geroni/igaf122.4002

**Published:** 2025-12-31

**Authors:** Nicolai Wohns, Sarah McKiddy

**Affiliations:** University of Washington, Seattle, Washington, United States; University of Washington, Seattle, Washington, United States

## Abstract

Healthspan is a central concept in aging studies and geroscience, typically equating health with the absence of disease or disability. We argue, however, that ‘healthspan’ rests on a narrow and often unexamined set of assumptions about what it means to be healthy and thereby marginalizes subjective and relational aspects of aging, especially the experiences of those living with chronic conditions, disabilities, or conditions outside normative biomedical models. This orientation risks pathologizing the experience of aging that may, in fact, be meaningful in important and underappreciated ways. The conventional definition of healthspan frames aging-related decline as categorically negative, which sidelines the potential for meaning, growth, or transformation within the experience of aging. It relegates illness and frailty to a deficit narrative rather than as conditions that can prompt reflection, deepen relationships, or cultivate virtues such as patience, humility, or resilience. In light of these limitations, we suggest a more comprehensive approach that deemphasizes the normalizing effect of healthspan, instead allowing what we value in life (e.g., flourishing, relational connection, or moral development) to take pride of place in shaping our metrics and priorities in geroscience.

